# Reading Fluency As a Predictor of School Outcomes across Grades 4–9

**DOI:** 10.3389/fpsyg.2017.00200

**Published:** 2017-02-14

**Authors:** Lucia Bigozzi, Christian Tarchi, Linda Vagnoli, Elena Valente, Giuliana Pinto

**Affiliations:** Department of Education and Psychology, University of FlorenceFlorence, Italy

**Keywords:** reading fluency, reading comprehension, school outcomes, school grades, predictors

## Abstract

This study analyzed the predictive relationship between reading fluency and school outcomes across school levels (primary, secondary, and high school), after controlling on the effect of reading comprehension. The sample included 489 children attending Italian primary (grades 4 and 5), secondary (grades 6 and 8), and high schools (grade 9). Students' reading fluency and comprehension were examined with a standardized reading achievement test. At the end of the school year, we requested the school reports of each participant. According to our data, reading fluency predicted all school marks in all literacy-based subjects, with reading rapidity being the most important predictor. School level did not moderate the relationship between reading fluency and school outcomes, confirming the importance of effortless and automatized reading even in higher school levels. Overall this study emphasizes the importance of identifying evidence-based tasks that can be administered in a short time and to many different individuals, which are easy to create, and are linked to school outcomes.

## Introduction

Teaching children to read fluently and comprehend a text is one of the main goals of early childhood education, because of the primary aims of reading which are to achieve one's goals, develop one's knowledge and potential, and participate in society (OECD, 2013). Reading is also a fundamental skill for school achievement (Hulme and Snowling, 2011), as also shown by studies documenting the persistence of reading disorders across the life span (Shaywitz et al., 1999). Reading fluency and comprehension are strictly inter-related, and also correlated with important aspects of academic life, such as school outcomes (Álvarez-Cañizo et al., 2015), or training success (Krumm et al., 2008). After primary school, teachers tend to focus on reading comprehension, neglecting the fostering of students' reading fluency, the influence of which is believed to fade on school outcomes. However, this assumption has recently been challenged, and the importance of reading fluency in adolescence re-evaluated (Rasinski et al., 2009; Ricketts et al., 2014; Zoccolotti et al., 2014). Moreover, recent literacy theories have documented how text use differs as a function of domains of academic subjects (Moje Birr et al., 2010), and reading strategies might become less generalizable as students move into increasingly specific disciplinary knowledge areas in higher school levels (Shanahan and Shanahan, 2008). This study analyzed the independent contribution of reading fluency to predict school outcomes in several subjects, after the effect of reading comprehension having been controlled for at three school levels, primary, secondary and high school in Italian students.

### The contribution of fluency to reading

Reading fluency is defined as the ability to read rapidly, accurately, and with the proper expression, and includes three main components, reading rapidity, accuracy, and prosody (Kuhn and Stahl, 2003; Álvarez-Cañizo et al., 2015; Elhassan et al., 2015). Although all three components play an important role for school achievement, the first two ones (i.e., rapidity and accuracy) are most commonly assessed, in both educational and clinical contexts. As an implication, the only standardized measures available, at least in the Italian context, are reading rapidity and accuracy tests. Hence, this study will focus on the effect of reading fluency and accuracy on school achievement.

Reading fluency represents an extremely complex process, as the reader has to integrate perceptual skills to automatically translate letters into coherent sound representations, lexical skills to unitize those sound components into recognizable wholes, and processing skills to identify meaningful connections within and between sentences, relate text information with prior knowledge, and make inferences to fill in the gaps in the text (Fuchs et al., 2001). These skills need to be coordinated in a seemingly effortless manner: reading fluency reflects this complex integration and can be used as a reliable measure of reading expertise. Indeed, effortless and efficient reading fluency frees up cognitive resources for the higher-level and demanding comprehension processing of the text (Fuchs et al., 2001). In this regard, the theoretical foundation is represented by seminal article LaBerge and Samuel's (1974). According to these authors, we are able to perform two or more activities (e.g., fluency and comprehension) if we alternatively direct our attention between the activities, or if one of the activities is so mastered that it is performed automatically. In non-fluent readers, attention is drained by the decoding activity, and cognitive resources are not available for the comprehending activity (Pikulski and Chard, 2005).

Certainly, reading, decoding and comprehension components are correlated (Pinnell et al., 1995; Pikulski and Chard, 2005). But decoding and comprehension can be separated, or at least dissociated. For example, when diagnosing a learning disorder with an impairment in reading, the DSM-V requires us to specify whether word reading accuracy, reading rate or fluency, spelling, or reading comprehension are compromised (American Psychiatric Association, 2013). The possibility that a compromised fluency might be dissociated from a compromised comprehension represents evidence that the two skills are lodged in distinct mechanisms. The relationship between reading fluency and comprehension is complex, and it is difficult to determine whether the former is a cause or a consequence of the latter, although several studies suggest that reading fluency influences the reading comprehension process (Fuchs et al., 2001; Rasinski et al., 2005; Nese et al., 2013).

### Reading fluency and school outcomes

Regarding these two skills, reading fluency has been neglected by studies, especially in later school levels. However, reading fluency is an important topic area of longstanding interest, and it is currently receiving considerable attention (Tichá et al., 2009; Ari, 2015; Elhassan et al., 2015). Reading fluency represents a crucial point for teachers to help struggling students meet school standards. Reading fluency becomes even more important in school settings based on learning from textbooks and time-limited assessment to determine students' outcomes. Indeed, many scholars consider reading fluency as a curriculum-based measurement, that is a valid and reliable procedure to monitor students' progress on a frequent basis and make instructional decisions (Tichá et al., 2009; Nese et al., 2013).

However, there is still disagreement on whether the magnitude of the correlation between reading fluency and high-stakes assessment scores declines across years (Reschly et al., 2009), or, instead, whether reading fluency is a key for successful school achievement even beyond elementary grades (Rasinski et al., 2005, 2009). This debate is still far from being settled, because the few studies conducted on this topic have led to contrasting results, neglecting the differences existing between languages (i.e., depth of orthography) and school system (i.e., school grades to measure students' outcomes). Regarding the first point, research on reading fluency growth is limited beyond grade 5 (Nese et al., 2013), and even those that explored the predictive power of reading fluency on school outcomes across grades lead to contradictory results (see Reschly et al.'s meta-analysis, 2009). Regarding the second point, learning to read in deep orthographies, characterized by an irregular mapping between letters and phonemes, is a much slower process than what happens in shallow orthographies, characterized by a regular mapping between letters and phonemes (Zoccolotti et al., 2008). In languages with a shallow orthography, such as Italian, reading accuracy is reached quite rapidly, making this parameter a less important indicator of reading proficiency and school outcomes than reading fluency (Bigozzi et al., 2016a,b). Thus, when assessing reading fluency, it is important to clearly distinguish the contribution of accuracy from that of rapidity. Regarding the second point, several studies have assessed students' school outcomes through standardized reading achievement tests. However, more recently some scholars have proposed school grades as a more ecological measure of school outcome. School grades have been criticized for low objectivity, reliability and validity (Krumm et al., 2008), but these issues have been criticized and disconfirmed by several studies that reported high correlations between school grades and other academic criteria (achievement tests, training success, and the like; Krumm et al., 2008; Rockoff and Speroni, 2010). A few authors even claimed that school grades might be a better predictor of graduation rates than standardized test scores, such as SAT scores (Bowen et al., 2009). School marks represent relevant real-life criteria to assess school outcomes (Krumm et al., 2008), and this is particularly true in countries in which school marks assigned by teachers represent the standard measure for students' school achievements and where students progress through school grades only if they have achieved at least a satisfactory level in each subject taught.

Finally, past research has demonstrated that comprehending and learning from text are associated but not overlapping processes (McNamara et al., 1996). Students might be able to achieve immediate comprehension of a text, but might not have learnt the concepts included in it. When students become metacognitively aware of the importance of reading comprehension for school, they begin to put more effort into this process. Non-fluent readers might invest most of their cognitive resources in comprehending a text, and this task might drain cognitive capacity from studying the text for school achievement. In this sense, reading comprehension might have a positive effect on school outcomes if reading fluency is effective, efficient and effortless. To the best of our knowledge, prior studies have not tested whether reading fluency mediates the relationship between reading comprehension and school outcomes or not.

### Aims of the study

The aim of this study was to analyze the predictive relationship between reading fluency and school outcomes across school levels (primary, secondary, and high school). More specifically, we expected that: (a) reading fluency contributes to predicting school marks in all school subjects in which reading plays a main role, after the effect of reading comprehension being controlled for; (b) reading fluency mediates the relationship between reading comprehension and school grades; (c) the contribution of reading fluency to school outcomes is not moderated by school level.

## Methods

### Participants

The sample included 489 children attending Italian primary (grades 4 and 5), secondary (grades 6 and 8), and high schools (grade 9) in a mid-sized city in Central Italy (see Table 1). From this sample we had previously excluded foreign children and those who were covered by a certificate attesting the presence of a Learning Disability. The parents of the participants gave informed consent for the participation of their children in the study. The measures were administered at a time agreed on with the school and with due adherence to the requirements of privacy and informed consent required by Italian law (Legislative Decree DL-196/2003). Regarding the ethical standards for research, the study referred to the last version of the Declaration of Helsinki (World Medical Association, 2013). The present study was approved by the Ethics Committee of the Department of Psychology at the University of Florence, Italy.

**Table 1:** **Sample description**.

**School**	**Grade**	***N***	**Males**	**Females**
Primary	4	143	77	66
	5	145	76	69
Secondary	6	70	34	36
	8	71	36	35
High	9	60	15	45
Total		489	238	251

In the Italian educational system, schools' programs are defined by the National Guidelines for the Curriculum, set by the Ministry of Education and Research. Students enter primary school at 6 years of age and stay for 5 years. Students enter secondary school at 11 years of age and this lasts for 3 years. At 14 years of age, students have to choose a specialization and enter high school, which lasts for 5 years. Class sizes are about 20 students in rural areas and small towns, and 30–35 students in large cities. The purpose of primary school is to teach the fundamental knowledge and skills to develop basic cultural competence. The subjects taught are: Italian, English, History, Geography, Mathematics, Science, Technology, Music, Art, and Physical Education. The timetable offers the following options: 24 h a week; 27 h a week; up to 30 h a week, involving up to 3 h per week for extra-curricular activities); or 40 h a week, including the lunchtime meal. In secondary school, the minimum teaching time is 30 h per week. The subjects taught are: Italian (9 h per week), in-depth studies in literary subjects (1 h per week), Mathematics and Science (6 h per week), Technology (2 h per week), English (3 h per week), second foreign language (2 h per week), Art (2 h per week), Physical Education (2 h per week) and Music (2 h per week). In high school the timetable is 27 h per week. The subjects taught depend on the specialization of the school, but all schools include: Italian, English, History, Geography, Mathematics, Science, Technology, Music, Art, and Physical Education. Period assessments take place twice every year, at the end of each 4-month term. The evaluations in each subject are the responsibility of the teacher and are expressed in numerical marks out of 10 (from 0 to 10)^1^.

### Measures

Students' reading fluency and comprehension were examined with a standardized reading achievement test (MT Reading Test, Cornoldi and Colpo, 1995, 2011; Cornoldi et al., 2010). These tests are standardized instruments currently used in Italy for the assessment of reading processes (fluency and/or comprehension). Their reliability and validity has been well established in both, the construction of the instrument, and in several studies conducted by multiple investigators (e.g., Levorato et al., 2004; Faccioli et al., 2008; Angelelli et al., 2010; Zoccolotti et al., 2014). For a more accurate sample selection, we also considered the score on the standardized reading achievement test, and excluded students whose performance in reading accuracy, fluency, and/or comprehension was lower than the 5th percentile, following the indications of the DSM-5 (American Psychiatric Association, 2013).

#### Reading fluency

Each participant was tested individually by a trained experimenter. The participant was required to read the passage according to his or her grade level. Instructions emphasized accuracy and speed (“Read aloud as accurately and rapidly as you can.”) while paying attention to the text content. The two components of reading fluency, rapidity (number of syllables read divided by time in seconds necessary to read them) and accuracy (number of words misread) were calculated. The following texts were assigned:

fourth grade, “L'indovina che indovinò” (“*The fortune-teller who guessed*,” 297 syllables);
fifth grade, “Vecchi proverbi” (“*Old sayings*,” 448 syllables);
sixth grade, “Sogni a Hiroshima” (“*Dreams in Hiroshima*,” 592 syllables);
eighth grade, “Città da salvare” (“*Cities to save*,” 576 syllables);
ninth grade, “26 Dicembre 2004” (“*26th December 2004*,” 1123 syllables).

#### Reading comprehension

The reading comprehension test was collectively administered by a trained experimenter. The participant had to silently read a text and answer multiple-choice questions, with the possibility of accessing the text. Texts, number of questions (10 or 15) varied with school levels. Raw scores were converted to z scores according to standard reference data (Cornoldi and Colpo, 1995, 2011; Cornoldi et al., 2010). The following texts were assigned:

fourth grade, “Il leone e la leonessa” (“The lion and the lioness,” 10 questions, e.g., “What is the savannah?” 241 words);
fifth grade, “Il viaggio delle anguille” (“The eels' journey,” 10 questions, e.g., “Which ocean do eels cross?” 267 words);
sixth grade, “Il pescatore, la volpe e l'orso” (“The fisherman, the fox, and the bear,” 15 questions, e.g., “Which of these sentences is more important for the development of the plot?” 503 words);
eighth grade, “Don Orione” (“Father Orione,” 15 questions, e.g., “Which word would you choose to substitute the term “herd”?” 193 words);
ninth grade, “Piaggia” (“*Piaggia*,” 15 questions, e.g., “Why was the rifle considered to be a prodigious weapon?” 333 words).

#### School marks

At the end of the school year, we requested the school reports of each participant, and took note of their scores in each subject (Italian, English as a foreign language, History, Geography, Mathematics, Sciences, Technology, Music, Art, and Physical Education). For most of the subjects we were able to collect data for all participants. As regards Technology, this subject is not taught in primary school, thus we collected data only for secondary and high school students, for a total of 234.

### Data analysis

Each variable's extreme outliers were identified and eliminated by observing the relative box-plots. Through examination of the skewness and kurtosis of each dependent variable's probability distribution we verified that all variables were normally distributed, except for reading accuracy. Thus, reading accuracy was normalized through a monotonic transformation.

The first hypothesis (independent contribution of reading fluency on school marks) were explored through a hierarchical multiple linear regression analysis, with reading comprehension included in the first step, reading fluency in the second step, and school marks as dependent variables.

The second hypothesis (mediational effect of reading fluency on the association between reading comprehension and school grades) was explored through a mediation analysis. The third hypothesis (moderating role of school level on the association between reading fluency and school marks) was explored through a moderation analysis. Both mediation and moderation analyses were conducted through PROCESS (release 2.15), an SPSS Macro created by Hayes (2012). The direct, indirect and moderation effects were derived from linear regression models. As suggested by Preacher and Hayes (2008), we used the bootstrapping strategy to test the mediation hypothesis, as it is the most powerful method to obtain confidence limits for specific indirect effects under most conditions. We used 5,000 bootstrap samples to construct bootstrap confidence intervals for indirect effect. The end points of bootstrap confidence intervals of the indirect effect were determined through the bias corrected method. In both tests, reading comprehension was included as a covariate. In the moderation analysis, the school moderating effect for each reading fluency component was tested by controlling the effect of the other component: when reading rapidity was the independent variable, we included reading accuracy as a covariate, and when reading accuracy was the independent variable, we included reading rapidity as a covariate.

## Results

Descriptive statistics for all the variables included in the study are reported in Table 2.

**Table 2:** **Descriptive statistics of all variables: participants, mean, minimum, maximum, standard deviation, skewness and kurtosis**.

	***N***	**Min**	**Max**	**M ± SD**	**Skewness**	**Kurtosis**
Reading fluency – rapidity (syllables/seconds)	489	1.21	7.48	4.25 ± 1.18	0.22 ± 0.11	−0.32 ± 0.22
Reading fluency – accuracy	487	0	18	2.12 ± 2.29	2.82 ± 0.11	12.49 ± 0.22
Reading comprehension	488	2	15	9.04 ± 0.18	−0.33 ± 0.11	0.59 ± 0.22
Italian	489	6	10	8.04 ± 1.14	−0.04 ± 0.11	−0.74 ± 0.22
English as a foreign language	489	6	10	8.10 ± 1.18	−0.13 ± 0.11	−0.81 ± 0.22
History	489	6	10	8.10 ± 1.24	−0.20 ± 0.11	−0.96 ± 0.22
Geography	489	6	10	8.02 ± 1.21	−0.17 ± 0.11	−0.92 ± 0.22
Mathematics	489	6	10	7.95 ± 1.20	−0.02 ± 0.11	−0.87 ± 0.22
Sciences	489	5	10	8.14 ± 1.18	−0.27 ± 0.11	−0.78 ± 0.22
Technology	387	6	10	8.19 ± 1.05	−0.11 ± 0.12	−0.59 ± 0.25
Music	489	6	10	8.36 ± 0.97	−0.19 ± 0.11	−0.20 ± 0.22
Art	489	6	10	8.50 ± 0.98	−0.25 ± 0.11	−0.30 ± 0.22
Physical Education	489	6	10	8.65 ± 0.91	−0.29 ± 0.11	−0.08 ± 0.22

The correlational analyses show that overall reading rapidity correlates positively with accuracy. The two reading fluency components are differently associated to reading comprehension. Reading rapidity and comprehension did not correlate at a statistically significant level, whereas reading accuracy and comprehension were negatively correlated: the fewer decoding mistakes made, the better the comprehension. Whereas reading rapidity and accuracy, and reading accuracy and comprehension correlated at all school levels, reading rapidity and comprehension correlated in grades 4, 5, 6, and 9, but not in grade 8 (see Table 3). Table 4 shows the correlation between reading measures and school outcomes for the total sample, as well as for each grade.

**Table 3:** **Correlational analysis of reading measures: rapidity, accuracy, and comprehension**.

	**Total**	**Grade 4**	**Grade 5**	**Grade 6**	**Grade 8**	**Grade 9**
Rapidity^*^Accuracy	−0.51^**^	−0.43^**^	−0.49^**^	−0.61^**^	−0.48^**^	−0.50^**^
Rapidity^*^Comprehension	0.05	0.43^**^	0.30^**^	0.38^**^	0.18	0.41^**^
Accuracy^*^Comprehension	−0.17^**^	−0.44^**^	−0.29^**^	−0.34^**^	−0.37^**^	−0.42^**^

* *p < 0.05*,

** *p < 0.01*.

**Table 4:** **Correlations between reading measures (rapidity, accuracy and comprehension) and school outcomes (Italian, English as a foreign language, History, Geography, Mathematics, Sciences, Technology, Music, Art, and Physical education) for the total sample (*n* = 489), and broken down by grade: 4 (*n* = 143), 5 (*n* = 145), 6 (*n* = 70), 8 (*n* = 71), and 9 (*n* = 60)**.

		**Ita**	**Eng**	**His**	**Geo**	**Mat**	**Sci**	**Tech**	**Mus**	**Art**	**P.E**.
Tot	Rap.	0.23^**^	0.24^**^	0.20^**^	0.22^**^	0.17^**^	0.20^**^	0.07	0.08	0.01	−0.05
	Acc.	−0.25^**^	−0.26^**^	−0.23^**^	−0.21^**^	−0.25^**^	−0.20^**^	−0.08	−0.14^**^	−0.09^*^	−0.05
	Comp.	0.38^**^	0.37^**^	0.41^**^	0.34^**^	0.31^**^	0.41^**^	0.27^**^	0.17^**^	0.24^**^	0.21^**^
Gr. 4	Rap.	0.40^**^	0.48^**^	0.44^**^	0.39^**^	0.35^**^	0.38^**^	0.26^**^	0.23^**^	0.19^*^	0.18^*^
	Acc.	−0.30^**^	−0.36^**^	−0.32^**^	−0.26^**^	−0.30^**^	−0.25^**^	−0.14	−0.24^**^	−0.24^**^	−0.15
	Comp.	0.55^**^	0.56^**^	0.61^**^	0.56^**^	0.51^**^	0.58^**^	0.42^**^	0.34^**^	0.33^**^	0.30^**^
Gr. 5	Rap.	0.47^**^	0.31^**^	0.43^**^	0.50^**^	0.43^**^	0.52^**^	0.38^**^	0.28^**^	0.17^*^	0.09
	Acc.	−0.33^**^	−0.24^**^	−0.32^**^	−0.32^**^	−0.31^**^	−0.34^**^	−0.22^*^	−0.18^*^	−0.22^**^	−0.15
	Comp.	0.32^**^	0.25^**^	0.33^**^	0.29^**^	0.35^**^	0.35^**^	0.25^**^	0.23^**^	0.19^*^	0.19^*^
Gr. 6	Rap.	0.61^**^	0.59^**^	0.42^**^	0.41^**^	0.38^**^	0.45^**^	0.30	0.18	0.47^**^	0.21
	Acc.	−0.44^**^	−0.45^**^	−0.43^**^	−0.38^**^	−0.44^**^	−0.36^**^	−0.21	−0.14	−0.21	−0.04
	Comp.	0.67^**^	0.61^**^	0.72^**^	0.63^**^	0.59^**^	0.63^**^	0.41^*^	0.26^*^	0.27^*^	0.28^*^
Gr. 8	Rap.	0.50^**^	0.57^**^	0.51^**^	0.53^**^	0.36^**^	0.45^**^	0.47^**^	0.58^**^	0.33^**^	−0.04
	Acc.	−0.23	−0.40^**^	−0.35^**^	−0.35^**^	−0.29^*^	−0.32^**^	−0.27^*^	−0.20	−0.09	−0.13
	Comp.	0.38^**^	0.28^*^	0.36^**^	0.37^**^	0.34^**^	0.25^*^	0.31^**^	0.12	0.11	0.08
Gr. 9	Rap.	0.78^**^	0.71^**^	0.63^**^	0.59^**^	0.40^**^	0.42^**^	0.41^**^	0.48^**^	0.44^**^	0.22
	Acc.	−0.55^**^	−0.51^**^	−0.39^**^	−0.38^**^	−0.33^*^	−0.32^*^	−0.39^**^	−0.42^**^	−0.37^**^	−0.35^**^
	Comp.	0.54^**^	0.53^**^	0.48^**^	0.50^**^	0.38^**^	0.43^**^	0.46^**^	0.50^**^	0.49^**^	0.41^**^

** p < 0.01;

* *p < 0.05. Gr., Grade; Tot., Total; Rap., Rapidity; Acc., Accuracy; Comp, Comprehension; Ita, Italian; Eng, English as a foreign language; His, History; Geo, Geography; Mat, Mathematics; Sci, Sciences; Tech, Technology; Mus, Music; P.E., Physical Education*.

The multiple regression analyses with hierarchical method showed that reading comprehension, inserted in step 1, significantly predicted school marks in all subjects (ΔR^2^ ranged from .03 in Music to .16 in History), whereas reading fluency, inserted in step 2, predicted only grades in Italian, English as a foreign language, History, Geography, Mathematics, and Sciences, with Δ*R*^2^ ranging between .03 and .06. In terms of specific contributions, Italian, English as a foreign language, and History were predicted by both components of reading fluency, Geography and Sciences were predicted by reading rapidity only, and Mathematics by reading accuracy only (see Table 5).

**Table 5:** **Results from the multiple regression analysis with hierarchical method to control for the effect of reading fluency on school grades**.

	**Step 1: RC**	**Step 2: RF**			**Total *R*^2^**
	**Δ*R*^2^**	**Δ*R*^2^**	**Rapidity (β)**	**Accuracy (β)**	
Italian	0.14^**^	0.05^**^	0.14^**^	−0.12^*^	0.19^**^
English as a foreign language	0.13^**^	0.06^**^	0.14^**^	−0.13^*^	0.18^**^
History	0.16^**^	0.04^*^	0.12^*^	−0.11^*^	0.20^**^
Geography	0.11^**^	0.04^*^	0.15^**^	−0.08	0.15^**^
Mathematics	0.09^**^	0.04^**^	0.05	−0.18^**^	0.13^**^
Sciences	0.16^**^	0.03^**^	0.13^**^	−0.07	0.19^**^
Technology	0.07^**^	0.00	0.04	−0.01	0.06^**^
Music	0.03^**^	0.01	−0.00	−0.11^*^	0.03^**^
Art	0.05^**^	0.01	−0.07	−0.10	0.05^**^
Physical Education	0.04^**^	0.01	−0.12^*^	−0.09	0.05^*^

* p < 0.05;

** *p < 0.01*.

*RC, Reading comprehension; RF, Reading Fluency; ns, non-significant*.

The contribution of reading comprehension to school outcomes was mostly direct, as the mediational effect of reading rapidity and/or accuracy were either non-significant (for Geography, Sciences, and Technology) or significant but marginal (for Italian, English, History, Mathematics, Music, Art, and Physical Education). Variances explained by partially mediated models ranged between 3 and 14% (see Table 6).

**Table 6:** **Results from the mediation analysis to control for the mediational effect of reading fluency (rapidity and accuracy) on the association between reading comprehension and school grades**.

	**Model**	**Total effect**	**Direct effect**	**Indirect effect of rapidity**			**Indirect effect of accuracy**		
	***R*^2^**	**β**	**β**	**β**	**BootLLCI**	**BootULCI**	**β**	**BootLLCI**	**BootULCI**
Italian	0.14^**^	0.20^**^	0.19^**^	0.004	−0.002	0.014	0.008^*^	0.001	0.021
English	0.13^**^	0.19^**^	0.18^**^	0.004	−0.001	0.014	0.009^*^	0.002	0.024
History	0.16^**^	0.23^**^	0.22^**^	0.003	−0.002	0.014	0.008^*^	0.001	0.022
Geography	0.11^**^	0.18^**^	0.17^**^	0.004	−0.003	0.016	0.006	−0.001	0.018
Mathematics	0.09^**^	0.17^**^	0.15^**^	0.001	−0.001	0.010	0.013^*^	0.004	0.028
Sciences	0.16^**^	0.22^**^	0.21^**^	0.004	−0.002	0.015	0.005	−0.001	0.017
Technology	0.07^**^	0.12^**^	0.12^*^	0.001	−0.001	0.009	0.001	−0.008	0.011
Music	0.03^**^	0.07^**^	0.07^**^	0.002	−0.005	0.003	0.004^*^	0.001	0.018
Art	0.05^**^	0.10^**^	0.10^**^	−0.002^**^	−0.009	0.001	0.006^*^	0.000	0.017
Physical Education	0.04^**^	0.08^**^	0.08^**^	−0.003	−0.011	0.001	0.005^*^	0.000	0.015

* p < 0.05;

** *p < 0.01. ns, non significant*.

Overall, school moderated the relationship between reading fluency and school outcomes, although the percentages of variance explained by the interaction between school and reading fluency were small (between 1 and 5%). For Italian, reading rapidity contributed to explaining variance in students' outcome in primary and high school, but not in secondary school. The analysis of confidence intervals, instead, did not confirm the moderation effect for reading accuracy. For English, reading rapidity contributed to explaining variance in students' outcome in primary and high school, but not in secondary school; reading accuracy contributed to explaining variance in students' outcome only in high school, but not in primary and secondary school. For history, reading rapidity contributed to explaining variance in students' outcome in primary and high school, but not in secondary school; reading accuracy contributed to explaining variance in students' outcome only in primary school, but not in primary or secondary school. For geography, reading rapidity contributed to explaining variance in students' outcome in primary and high school, but not in secondary school. For mathematics, school did not moderate the relationship between reading fluency and students' outcome. For sciences, reading rapidity contributed to explaining variance in students' outcome in primary school, but not in secondary and high school. For music, reading accuracy contributed to explaining variance in students' outcome in high school, but not in primary or secondary school. For physical education, reading rapidity contributed to explaining variance in students' outcome in secondary school, but in primary or high school (see Table 7).

**Table 7:** **Results from the moderation analysis to control for the effect of school level on the association between reading fluency and school grades, with reading comprehension as a covariate, reading accuracy as a covariate for reading rapidity, and reading rapidity as a covariate for reading accuracy (*R*^2^, *R*^2^ change, unstandardized regression coefficients, lower and higher confidence intervals)**.

**DV**	**IV**	**Model**	**Interaction**	**Primary**			**Secondary**			**High**		
		***R*^2^**	**Δ*R*^2^**	**β**	**LLCI**	**UCLI**	**β**	**LLCI**	**UCLI**	**β**	**LLCI**	**UCLI**
Italian	Rap.	0.36^**^	0.05^**^	0.25^**^	0.13	0.38	0.05	−0.10	0.18	1.13^**^	0.82	10.45
	Acc.	0.34^**^	0.03^**^	−0.46^*^	−0.92	−0.01	0.79^*^	0.17	1.40	−1.91^**^	−2.98	−0.84
English	Rap.	0.31^**^	0.03^**^	0.25^**^	0.12	0.38	0.09	−0.06	0.24	0.97^**^	0.63	1.31
	Acc.	0.29^**^	0.02^**^	−0.45	−0.94	0.04	0.34	−0.32	1.00	−1.70^**^	−2.86	−0.55
History	Rap.	0.33^**^	0.03^**^	0.31^**^	0.17	0.45	−0.02	−0.18	0.14	0.67^**^	0.32	1.02
	Acc.	0.32^**^	0.01^**^	−0.59^*^	−1.09	−0.08	0.64	−0.03	1.32	−0.61	−1.79	0.57
Geography	Rap.	0.33^**^	0.03^**^	0.37^**^	0.24	0.50	−0.02	−0.18	0.14	0.63^**^	0.28	0.97
Maths	Acc.	0.27^**^	0.00	−0.67^**^	−1.18	−0.16	−0.13	−0.82	0.55	−0.82	−2.02	0.36
Sciences	Rap.	0.27^**^	0.02^**^	0.33^**^	0.19	0.46	−0.04	−0.20	0.12	0.35	−0.01	0.70
Music	Acc.	0.21^**^	0.02^**^	−0.27	−0.69	0.16	0.28	−0.30	0.85	−1.45^**^	−2.45	−0.45
P.E.	Rap.	0.12^**^	0.03^**^	−0.02	−0.14	0.09	−0.34^**^	−0.47	−0.21	−0.01	−0.30	0.29

** *p < 0.01*,

* *p < 0.05. Rap., Rapidity; Acc., Accuracy; P. E., Physical Education*.

## Discussion

The aim of this study was to analyze whether reading fluency influences students' school outcomes in school subjects, independently of the effect of reading comprehension, and whether the independent contribution of reading fluency is moderated by the school level. According to the correlational analysis, reading fluency and comprehension are associated, confirming prior studies (Pinnell et al., 1995; Pikulski and Chard, 2005). More specifically, reading accuracy appears to be more strictly associated with reading comprehension than reading rapidity, confirming that reading “fast” does not help children to adequately process the information included in the text. It is important to address a deep form of reading fluency, according to which this construct is part of a developmental process of building decoding skills that are reciprocally and causally connected with reading comprehension, rather than just be considered as “fast reading” (Pikulski and Chard, 2005).

This study emphasizes the fundamental importance of reading comprehension and fluency in students' school outcomes. Reading comprehension and fluency are strictly inter-related processes, however, according to our data, both contribute independently to school marks in several subjects.

Firstly, although several studies suggest that reading fluency influences the reading comprehension process (Fuchs et al., 2001; Rasinski et al., 2005; Nese et al., 2013), results from the mediational analyses showed that the contribution of reading comprehension to school marks is mostly direct. More importantly, this study contributes to re-evaluating the role played by reading fluency, and confirms that effortless and automatic reading fluency frees up important cognitive resources for the comprehension activity, a high-level and demanding process (Fuchs et al., 2001; Pikulski and Chard, 2005; Tichá et al., 2009; Nese et al., 2013). The efficacy of reading fluency is especially significant for subjects in which literacy skills and textbook studying play a primary role (i.e., Italian, English, History, Geography, Mathematics, and Sciences). Reading rapidity was the most important predictor among the two reading fluency components: as suggested by several scholars, in shallow orthographies, reading accuracy is reached rapidly, which makes reading rapidity a much more important indicator of reading proficiency (Zoccolotti et al., 2008; Pinto et al., 2015). Instead, reading accuracy played an important role for Geography and Mathematics, probably as these subjects involve more focused attention on visuo-spatial elements, besides the verbal one (Schnotz, 2002). Many textbooks require students to mostly process verbal information, whereas in Geography and Mathematics, students need to integrate verbal and graphic information, an activity that requires a slower and more accurate processing (Massey and Riley, 2013).

The moderation analysis contributed to our understanding of the relationship between reading fluency and school outcomes. Overall, the effect was mainly confirmed for primary school, when students are in the process of reading acquisition (Pinto et al., 2015). In secondary school instead, reading fluency appears to be neglected, except for Italian in which there is still a strong emphasis on grammar. In secondary school reading fluency did not influence students' outcomes. The importance of reading fluency for school outcomes in primary school is not questionable, since the teachers' focus at this level is on basic literacy skills (Firestone and Herriott, 1982; Alvermann and Moore, 1991). Once in secondary school, the focus shifts to subject-matter literacy (Knott, 1986; Alvermann and Moore, 1991). However, secondary school instruction mainly puts emphasis on factual textual information, with textbooks acting as sources of information (Smith and Feathers, 1983; Alvermann and Moore, 1991). Consequently, students do not need to dedicate many cognitive resources to the reading comprehension process, which also allows poor decoders to achieve good school outcomes. Instead, at high school level, reading fluency brings again an independent contribution to school outcomes. Several reasons are able to explain this result: (i) in high school basic literacy skills are no longer sufficient, because of the complexity of textbooks (Lester and Cheek, 1997); (ii) students who enter high school with a lack of sufficient reading fluency are not likely to find instructional support from teachers and/or remedial programs (Rasinski et al., 2005; Joseph and Schisler, 2009), and (iii) slow readers require significantly more time in accomplishing school tasks than normally-reading readers do, which might eventually lead to frustration, task-avoidance behaviors, and school failure (Rasinski et al., 2005; Archer et al., 2013). Overall, these results confirm the importance of reading fluency even in adolescence (Rasinski et al., 2009; Ricketts et al., 2014; Zoccolotti et al., 2014).

Overall this study emphasizes the importance of identifying evidence-based tasks that can be administered in a brief time and by many different individuals, which are easy to create, and are linked to school outcomes (Tichá et al., 2009; Nese et al., 2013). As the effect of reading fluency on school outcomes does not fade after primary school, secondary and high school teachers should not underestimate the negative impact of ineffective and non-automatic reading fluency has on students' learning. This study also contributes to extend what we know about learning disorders on normally-developing children. In shallow orthographies, dyslexic readers have compromised accuracy and reading rapidity (Bigozzi et al., 2016a), and these compromised processes hinder student learning. This study confirms the same effect for the population of students without a learning disorder.

The results of this study are affected by a few limitations. A few intervening variables might explain the relationship between reading and learning, both higher-order (e.g., studying skills, metacognitive variables, or motivational variables, see Schiefele et al., 2012), and lower order ones (e.g., verbal ability, see Tilstra et al., 2009). Future studies should also include these variables in the research design to better explain under which conditions reading fluency fosters students' learning and their school outcomes. Moreover, although cross-sectional data can be used to test mediation, longitudinal data are more appropriate. Future studies should replicate the results of this study through a longitudinal research design. Finally, few authors have emphasized the importance of reading prosody, besides rapidity and accuracy, for school achievement (Kuhn and Stahl, 2003). However, there is a lack of standardized measures of this reading fluency component, and future studies should aim at first validating reading prosody assessment and then analyzing the specific contribution of this component on school achievement.

Reading fluency is typically considered an important process for school achievement in beginning readers and in dyslexic students. This study provides new insights into the importance of fluent reading for academic outcomes beyond reading comprehension. The shift from reading comprehension (more common in fluency research) to academic performance as the criterion variable in the study is novel and yielded important findings for the field. Our results contribute to renew the attention to specific processes (in this case, reading fluency) for school achievement, besides more general processes (such as intelligence), which can also be improved as a result of targeted interventions.

## Author contributions

All authors listed, have made substantial, direct and intellectual contribution to the work, and approved it for publication.

### Conflict of interest statement

The authors declare that the research was conducted in the absence of any commercial or financial relationships that could be construed as a potential conflict of interest.

## Footnotes

^1^www.indire.it/lucabas/lkmw_img/eurydice/quaderno_eurydice_30_per_web.pdf
